# Molecular mechanisms of amyloid inhibition: an NMR-driven framework with polyphenols as a case study

**DOI:** 10.3389/fmolb.2025.1676927

**Published:** 2025-09-12

**Authors:** Giacomo Zuccon, Aakriti Darnal, Edoardo Longo, Sara D’Aronco, Emanuele Boselli, Patrick Orlando, Alberto Ceccon

**Affiliations:** ^1^ Laimburg Research Centre, Vadena, Italy; ^2^ Faculty of Agricultural, Environmental and Food Sciences, Free University of Bozen-Bolzano, Piazza Università, Bolzano, Italy; ^3^ Department of Life and Environmental Sciences, Polytechnic University of Marche, Ancona, Italy

**Keywords:** amyloid aggregation, intrinsically disordered proteins, polyphenols, solution-state NMR spectroscopy, kinetic modeling, protein aggregation inhibitors, Oligomeric Intermediates, Huntington’s disease

## Abstract

Misfolding and aggregation of intrinsically disordered proteins into amyloid fibrils are central to neurodegenerative diseases such as Parkinson’s, Alzheimer’s, and Huntington’s. Increasing evidence suggests that transient, low-populated oligomeric intermediates, rather than mature fibrils, are key cytotoxic species. Natural polyphenols have shown promise as amyloid inhibitors, though their mechanisms of action remain unclear due to the complexity of early aggregation. This perspective explores how solution-state NMR can quantitatively assess inhibitor mechanisms. Building on recent literature elucidating the aggregation mechanisms of the huntingtin exon 1 protein (htt^ex1^), responsible for Huntington’s disease, we propose a kinetic framework that integrates early reversible oligomerization with downstream fibril formation and models the impact of small-molecule binding at distinct stages of the pathway. We show that monomer sequestration and inhibition of elongation-competent nuclei produce distinct aggregation profiles, resolvable through global fitting of NMR and kinetic data. This mechanistic insight enables classification of inhibitors by target stage—monomeric, oligomeric, or fibrillar—and demonstrates how polyphenols serve as a biologically relevant case study for applying this general NMR-driven framework to the design of small-molecule amyloid inhibitors.

## 1 Introduction

Protein misfolding, aggregation, and fibrillation are central to neurodegenerative diseases such as Parkinson’s, Alzheimer’s, and Huntington’s. These processes are often initiated by partial unfolding of native monomeric precursors, generating aggregation-prone conformers that nucleate the formation of toxic oligomers and, ultimately, insoluble cross-β sheet fibrils (Chen and Wetzel, 2001; Fitzpatrick et al., 2013; Sawaya et al., 2007).

While mature amyloid fibrils were historically considered the primary pathogenic entities, accumulating evidence identifies early-stage intermediates—monomers, dimers, low-molecular-weight oligomers, and protofibrils—as the principal mediators of cellular toxicity (Cascella et al., 2021; Fusco et al., 2017; Li et al., 2011; Miguez et al., 2023; Nucifora et al., 2012). Consequently, early misfolded intermediates represent critical targets for therapeutic intervention, offering a window of opportunity before irreversible fibril deposition (Chebaro and Derreumaux, 2009; Garfagnini et al., 2024; Lemkul and Bevan, 2012).

Despite this understanding, most current therapeutic strategies remain symptomatic and lack the molecular precision to address upstream misfolding and oligomerization events. Therefore, there is a pressing need to identify inhibitors that can selectively target transient, low-populated species to delay or prevent pathology at its origin. Shifting the focus from downstream fibrillar insoluble aggregates to early soluble species may enable effective disease-modifying treatments and redefine the therapeutic paradigm for amyloid-related disorders.

In this context, solution-state nuclear magnetic resonance (NMR) spectroscopy stands out as a powerful tool. Unlike fluorescence-based assays such as Thioflavin T (ThT), which selectively binds β-sheet-rich fibrils (Biancalana and Koide, 2010), or more recent-developed probes like ANS and taBODIPY designed to detect pre-fibrillar intermediates (Li et al., 2024), but still constrained by extrinsic binding, limited structural insight, and potential interference with native aggregation pathways—solution NMR enables atomic-resolution analysis of protein conformational states and interactions.

**FIGURE 1:**
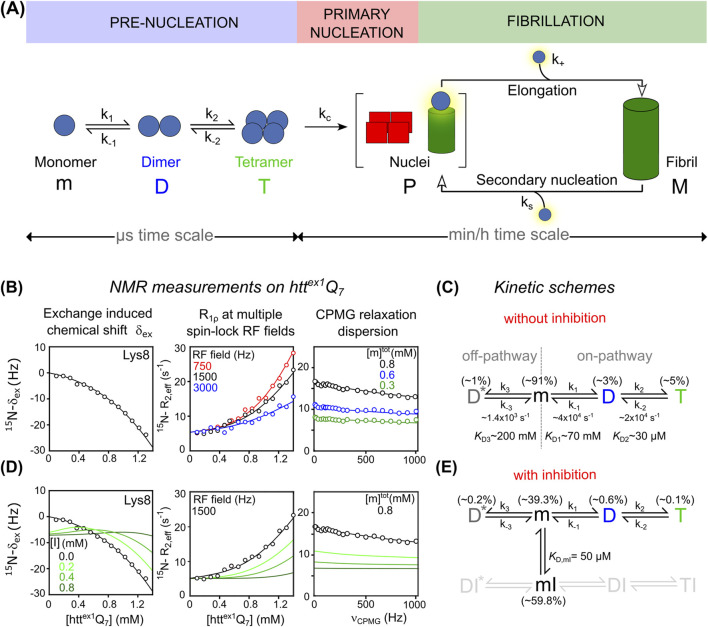
**(A)** Schematic representation of the aggregation pathway of the pathogenic htt^ex1^Q_35_ variant, including pre-nucleation events, primary nucleation, and fibril elongation. Monomeric htt^ex1^ (m) sequentially self-associates into tetrameric helical bundles (T) via coiled-coil dimers (D), followed by irreversible conversion into elongation-competent nuclei (P), which seed fibril formation (M). **(B)** NMR measurements used to probe pre-nucleation oligomerization of non-pathogenic htt^ex1^Q_7_: exchange-induced chemical shift perturbation (δ_ex_) acquired as a function of concentration of htt^ex1^Q_7_, concentration-dependent ^15^N R_1_ρ relaxation dispersion profiles recorded at three spin-lock RF field strengths: 750 Hz (red), 1,500 Hz (black), and 3,000 Hz (blue), ^15^N CPMG relaxation dispersion curves acquired for [htt^ex1^Q_7_] = 0.3 mM (green), 0.6 mM (blue), and 0.8 mM (black). In all 3 panels, best-fitted curves and experimental data are represented as solid lines and circles, respectively. Data adapted from (Ceccon et al., 2022; Ceccon et al., 2020a). **(C)** Corresponding kinetic scheme that accounts for experimental data, describing the pre-nucleation of htt^ex1^Q_7_. The off-pathway dimer species (D*) is shown here for completeness. **(D)** Effects of inhibitor on htt^ex1^Q_7_ pre-nucleation and corresponding NMR observables. Simulated δ_ex_, R_1_ρ and CPMG data in the presence of increasing concentrations of inhibitor ([I] = 0.2, 0.4, 0.8 mM) are based on the extended kinetic model in panel **(E)** where the inhibitor selectively binds monomeric htt^ex1^Q_7_ (m) forming a reversible monomer-inhibitor complex (mI) ( $K_{D,mI}$ = 50 μM). Additional details of the simulations of δ_ex_, R_1_ρ, and CPMG data are provided in the Supplementary Material (SI). Species distributions (D*, m, D, T*,* mI) shown in **(C)** and **(E)** correspond to the equilibrium populations calculated for [htt^ex1^Q_7_] = 1.2 mM.

Here, we highlight recent NMR advances that enable the detection and kinetic characterization of transient species exchanging on the microsecond-to-millisecond timescale, alongside the detailed elucidation of early aggregation mechanisms. We focus on huntingtin exon 1 protein (htt^ex1^) as a model amyloid system. Its well-characterized aggregation behavior and defined oligomerization intermediates make it ideally suited for dissecting amyloid nucleation and fibrillation mechanisms (Ceccon et al., 2020b; Ceccon et al., 2022; Kotler et al., 2019). As shown in Figure 1A, htt^ex1^ aggregation proceeds via a rapid “pre-nucleation” phase that generates elongation-competent nuclei through “primary nucleation”. These nuclei seed fibril elongation by monomer addition and can also catalyze “secondary nucleation” on fibril surfaces, thereby amplifying the aggregation process. This sequence ultimately leads to the accumulation of insoluble amyloid fibrils during the slower “fibrillation” phase (Ceccon et al., 2022; Torricella et al., 2024).

We further introduce a theoretical kinetic framework extending classical aggregation models (Cohen et al., 2011c; Cohen et al., 2011a; Cohen et al., 2011b) by incorporating reversible inhibitor binding at distinct stages of the aggregation process. With this perspective, we aim to provide a conceptual and methodological platform for the rational design and screening of inhibitors targeting nucleation intermediates in amyloid-related disorders.

### 1.1 Natural polyphenols as potential inhibitors of amyloid aggregation

Among the various classes of small molecules studied as aggregation inhibitors, natural polyphenols have emerged as broad-spectrum modulators of amyloid formation. Numerous studies support their neuroprotective effects across models of neurodegenerative diseases. For instance, resveratrol, a stilbene from grapes, has shown protective activity in Alzheimer’s models (Ge et al., 2012; Ladiwala et al., 2010). Taxifolin, a dihydroflavonol, inhibits Aβ oligomers (Sato et al., 2013), while (−)-epigallocatechin-3-gallate (EGCG), found in grape seeds and green tea, interferes with β-sheet formation and reduces mutant huntingtin aggregation (Ehrnhoefer et al., 2011; Fernandes et al., 2021). Among simpler phenolics, gallic acid—a major grape-derived compound—has shown significant anti-amyloidogenic activity. It inhibits aggregation of both Aβ and α-synuclein, shifting the equilibrium toward off-pathway, non-toxic oligomers (Liu et al., 2014; 2013).

Despite these promising outcomes, the precise molecular mechanisms of polyphenol-mediated inhibition remain unclear. Evidence suggests polyphenols interact with conserved structural motifs that recur across amyloidogenic proteins (Barreca et al., 2017; Ehrnhoefer et al., 2011; Murray et al., 1994). Polyphenols offer attractive scaffolds due to their chemical diversity, modifiability, and tunable bioavailability (Sahraeian et al., 2024; Williamson, 2025), allowing for structure–activity relationship studies and synthetic optimization (Hasnat et al., 2024; Liu et al., 2024). This versatility, combined with natural origin and low toxicity, makes them ideal leads for developing selective aggregation inhibitors (Davison and Brimble, 2019).

## 2 Probing amyloid pre-nucleation, fibril formation and inhibition mechanisms via NMR

### 2.1 Exchanged-induced chemical shift and relaxation techniques

Understanding the molecular events that precede amyloid fibril formation is essential to identify early determinants of protein misfolding diseases. Pre-nucleation processes typically involve low-populated, short-lived oligomeric species, often invisible to conventional structural biology tools. Yet in systems such as Aβ, α-synuclein, huntingtin, and tau, such elusive intermediates play critical roles in seeding aggregation, modulating toxicity, and shaping the pathway toward mature fibrils (Fusco et al., 2017; Li et al., 2011; Miguez et al., 2023; Nucifora et al., 2012). Solution NMR methods—including Carr–Purcell–Meiboom–Gill (CPMG) relaxation dispersion (Palmer, 2004), exchange-induced chemical shift (δ_ex_) (Vallurupalli et al., 2011), and spin-lock R_1ρ_ measurements (Palmer and Massi, 2006)—have enabled detection and kinetic characterization of these elusive species. These techniques were systematically applied to huntingtin exon 1 (htt^ex1^) protein by the group of Marius G. Clore (Ceccon et al., 2020b; Ceccon et al., 2020a; Ceccon et al., 2021a; Ceccon et al., 2022; Kotler et al., 2019), establishing a robust experimental and analytical framework for quantifying pre-nucleation oligomerization. Htt^ex1^ comprises three domains: an N-terminal amphipathic segment (NT, ∼17 residues) that promotes oligomerization and aggregation; a central polyglutamine (polyQ) tract, whose pathological expansion (≥35 glutamines) triggers rapid aggregation and amyloid fiber formation, ultimately leading to neuronal dysfunction; and a proline-rich domain (PRD) which reduces aggregation by increasing solubility (Bates et al., 2015; Chen and Wetzel, 2001; Kar et al., 2011).

To dissect early molecular events of amyloid aggregation, Clore’s group employed a non-pathological htt^ex1^Q_7_ construct containing only 7 glutamine repeats (Ceccon et al., 2020b; Ceccon et al., 2021a). This truncated construct remains largely monomeric for several weeks, enabling high-resolution NMR studies under near-physiological conditions. To investigate the mechanism of pre-nucleation oligomerization, a combination of NMR experiments was conducted on htt^ex1^Q_7_ at 5 °C, including ^15^N and ^13^Cα CPMG relaxation dispersion, exchange-induced chemical shift (^15^N and ^13^Cα δ_ex_) analyses, and ^15^N R_1ρ_ relaxation rates in the rotating frame obtained at several spin-lock field strengths. Measurements were performed at various protein concentrations and magnetic fields, enabling global kinetic modeling (Figure 1B).

Results revealed a branched oligomerization model with two competing pathways (Figure 1C). In the on-pathway, htt^ex1^ monomers (m) first form coiled-coil dimers (D), which then assemble into tetrameric helical bundles (T), a process driven by the NT segment. In parallel, an off-pathway yields non-productive dimers (D*) that cannot proceed further. Exchange between m and D* occurs on a slower timescale (∼750 μs), well captured by ^15^N and ^13^Cα CPMG dispersion. In contrast, the faster monomer-tetramer exchange (∼50 μs) was primarily captured through δ_ex_ and ^15^N R_1ρ_. Dissociation constants describing the equilibrium m ⇌ D, m ⇌ D*, and D ⇌ T yield values of ∼70 mM, ∼200 mM and ∼30 μM, respectively. At 1.2 mM total htt^ex1^Q_7_, populations were ∼1% for D*, ∼3% for D, and ∼5% for T.

These findings underscore the remarkable sensitivity of NMR relaxation techniques in detecting and quantifying transient, low-populated oligomers that elude conventional structural methods, offering a powerful approach to unravel early amyloid aggregation at atomic resolution.

The impact of inhibitors, such as polyphenols, on htt^ex1^Q_7_ pre-nucleation can be assessed by NMR. Only the binding between the inhibitor (I) and the monomer (m) is kinetically accessible, as the low populations of D and T (≤5%) and their rapid exchange rates (10–100 μs) preclude equilibrium with ligands that generally bind on the ms-to-s timescale.

Although traditional NMR and/or ITC experiments are generally sufficient to extract the equilibrium dissociation constant, $K_{D,mI}$ and stoichiometry of the inhibitor–monomeric htt^ex1^ (mI) complex, concentration-dependent ^15^N and ^13^Cα δ_ex_ and ^15^N R_1ρ_ relaxation rates measurements are needed to probe partial or complete inhibition of the fast monomer–tetramer exchange. As shown from simulations in Figure 1D, the binding of an inhibitor (assuming concentrations of [I] = 0.2, 0.4 and 0.8 mM and $K_{D,mI}$ = 50 µM consistent with previous literature (Ahmed et al., 2017; Marcinko et al., 2020)) results in a suppression of concentration-dependent δ_ex_ shifts as well as a significant reduction in R_1ρ_ rates and CPMG RD dispersion (additional details are provided in the SI). As highlighted in the corresponding kinetic scheme in Figure 1E, this reflects a decreased population of m, D, and T, consistent with inhibition of the productive tetramerization pathway (i.e., for [I] = 0.8 mM and [htt^ex1^Q_7_] = 1.2 mM, ∼0.2% for D*, ∼1.5% and 2% for D and T, respectively).

Note that for the purposes of this perspective, we are considering only inhibitors that effectively block the productive pathway (shown in gray in Figure 1E), as reversible tetramerization is tightly linked to the formation of elongation-competent nuclei (P) and represents a critical early step in amyloid fibril formation (M) (see Figure 1A). For further discussion of the m ⇌ mI equilibrium and modeling assumptions, see SI.

Given the limited relevance of the non-productive (m ⇌ D*) pathway to fibril formation, an ideal inhibitor might selectively target the productive exchange while leaving the mI ⇌ DI* equilibrium largely unaffected, therefore without a selective quenching of CPMG dispersion as described for the binding of Profilin to monomeric htt^ex1^ (Ceccon et al., 2020b).

### 2.2 Time-resolved NMR techniques

**FIGURE 2:**
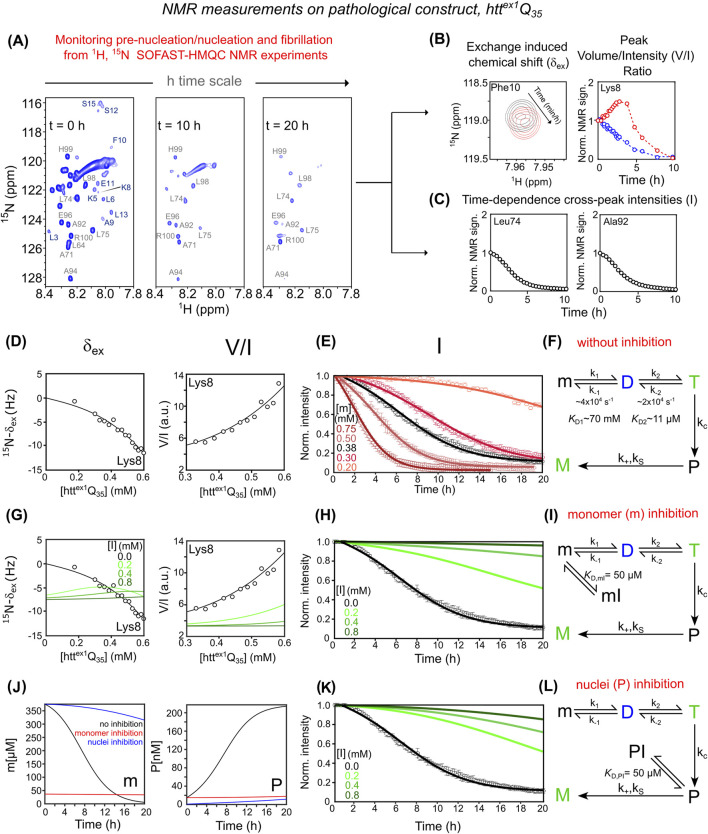
Mechanistic dissection of htt^ex1^Q_35_ aggregation and inhibition via NMR and kinetic modeling. **(A)** Aggregation monitored by ^1^H-^15^N SOFAST-HMQC spectra recorded at t = 0 h, 10 h, and 20 h **(B)** NMR observables reporting pre-nucleation oligomerization of htt^ex1^Q_35_: (left) exchange-induced ^15^N-δ_ex_ for Phe10 at t = 0 h (black) and 10 h (red); (right) time-dependent evolution of peak volume (in blue) and intensity (in red) for Lys8. **(C)** Time-dependent decay of ^1^H-^15^N cross-peak signal intensities for PRD residues Leu74 and Ala92, indicating irreversible fibril growth. **(D)** Pre-nucleation analysis: (left) ^15^N-δ_ex_, and (right) peak V/I ratio for Lys8 as a function of htt^ex1^Q_35_ concentration. **(E)** Aggregation kinetics of htt^ex1^Q_35_ monitored by PRD signal decay, acquired at increasing protein concentrations (0.20–0.75 mM). **(F)** Kinetic model that accounts for experimental data describing htt^ex1^Q_35_ aggregation without inhibitor. The model integrates reversible pre-nucleation equilibria (m ⇌ D ⇌ T), irreversible conversion of tetramers into elongation-competent nuclei (T → P) governed by, and subsequent fibril elongation (P → M). In **(D)** and **(E)**, best-fitted curves and experimental data are represented as solid lines and circles, respectively. **(G)** Effect of monomer sequestration on concentration-dependent δ_ex_ (left) and V/I data (right) for Lys8 across different inhibitor concentrations. **(H)** PRD signal decay kinetics under monomer inhibition. Green curves in **(G)** and **(H)** show simulations of monomer inhibition at increasing concentrations ([I] = 0, 0.2, 0.4, 0.8 mM) based on the extended model described in **(I)** which includes reversible binding of the inhibitor to the monomer (m ⇌ mI, with K_D,mI_ = 50 μM). **(J)** Simulated time-course of monomeric htt^ex1^Q_35_ “m” (left panel) and elongation-competent “P” nuclei (right panel) in the absence (black) and in the presence of inhibitor ([I] = 0.8 mM and [m] = 0.38 mM). Red and blue curves represent inhibition by sequestration of m and P, respectively. **(K)** PRD signal decay kinetics under nucleus sequestration. Green curves in **(K)** represent simulations at increasing inhibitor concentrations ([I] = 0, 0.2, 0.4, 0.8 mM) based on the extended model described in **(L)**, which incorporates reversible binding of the inhibitor to the nuclei (P ⇌ PI, with *K*_*D,PI*_ = 50 μM). **(A)** And data shown in **(B–E,G,H,K)** are adapted from (Ceccon et al., 2022; Torricella et al., 2024).

Time-intensive relaxation-based NMR experiments used for htt^ex1^Q_7_ are not applicable to pathological htt^ex1^Q_35_, as monomer signals decay rapidly due to aggregation over a few hours (Ceccon et al., 2021; Ceccon et al., 2022). Nonetheless, detailed insight into pre-nucleation, nucleation, and fibrillation can be achieved by acquiring time-resolved 2D ^1^H–^15^N SOFAST-HMQC (Schanda et al., 2005) spectra (Figure 2A).

Rapid pre-nucleation transitions (m ⇌ D ⇌ T) are characterized through time-dependent δ_ex_ (Figure 2B, left), similarly to what was previously described for the non-pathological htt^ex1^Q_7,_ and ^1^H–^15^N cross-peak volume/intensity (V/I) ratios of residues in the NT segment (Figure 2B, right). Off-pathway dimerization (m ⇌ D*) contributes negligibly to δ_ex_ and V/I and is irrelevant to fibril formation; it is thus excluded. As shown in Figure 2B, for these NT residues, cross-peak volume and intensity diverge during the early phase: cross-peak volume decreases from *t = 0*, while intensity initially increases, peaking at 2–3 h before declining. This reflects chemical exchange line broadening caused by rapid tetramerization (with significant ^15^N and ^1^H_N_ shift differences between monomeric and transient oligomeric species). Since tetramer population (T) scales with [m]^3^, an initial drop in monomer reduces line broadening, temporarily narrowing peaks and increasing intensity—until monomer depletion dominates and the signal fades.

In contrast, slow fibril formation kinetics are extracted from the gradual decay of PRD ^1^H–^15^N intensities (Figure 2C), a region not subject to fast exchange, serving as a clean readout for irreversible aggregation. This dual-observable strategy enhances the mechanistic understanding of aggregation-prone htt^ex1^Q_35_ and enables characterization of inhibitor effects.

Global fitting of δ_ex_ and V/I data to the m ⇌ D ⇌ T model (Figures 2D,E) shows that the m ⇌ D equilibrium constant (*K*_D1_ ∼ 70 mM) matches that for htt^ex1^Q_7_. In contrast, the D ⇌ T constant (*K*_D2_ ∼11 μM) is 2–3 times smaller for htt^ex1^Q_35_, indicating increased tetramer stability (∼0.4–0.6 kcal/mol). The simplest kinetic scheme that accurately fits data in Figure 2F includes primary nucleation, secondary nucleation, and elongation. This aligns with the updated kinetic scheme proposed by Clore’s group (Torricella et al., 2024), where tetramers formed rapidly on the μs timescale convert irreversibly to elongation-competent nuclei (P), which then seed fibrillation (M) via elongation and secondary nucleation (see SI for additional details and derivation).

Given the mechanism of htt^ex1^Q_35_ aggregation and according to differential Supplementary Equations S1, S2, inhibition of fibril formation can occur at distinct stages:

1. Sequestration of m into mI shifts the pre-nucleation equilibrium (mI ⇌ m ⇌ D ⇌ T) away from higher-order oligomers. This effect is reflected in changes to concentration-dependent δ_ex_ and V/I ratios (Figure 2G) in simulations with [I] = 0.2, 0.4, 0.8 mM, assuming *K*_D,mI_ = 50 µM. Reduced availability of free monomer (m_free_ ≪ m_tot_) lowers the formation rate of elongation-competent nuclei (P), thereby suppressing both secondary nucleation (second term in Supplementary Equation S1) and elongation (Supplementary Equation S2). These effects manifest as a slower decay of PRD signal intensities with increasing [I] (Figure 2H). The corresponding kinetic model incorporating the off-pathway “mI” state is shown in Figure 2I, with time-dependent evolution of m(t) and P(t) illustrated in Figure 2J.
2. Sequestration of P nuclei into PI affects the time course of PRD intensity decay (Figure 2K), without altering δ_ex_ or V/I, since P forms downstream of the m ⇌ D ⇌ T equilibrium. A significant reduction in free P (P_free_ ≪ P_tot_) impairs fibril elongation (Supplementary Equation S2), as represented in the kinetic model including the PI state (Figure 2L). Although P is a monomeric unit at the oligomer end, its conformation and environment may differ from that of the free monomer, potentially exposing distinct ligand-binding surfaces. Similar mechanisms have been observed for tau, Aβ, and actin, where inhibitors cap fibril ends by recognizing polymerization-competent termini. Thus, selective binding to the P state is both structurally plausible and biologically relevant. (Bai et al., 2025).

Importantly, although both mechanisms initially produce similar inhibition profiles due to pre-existing elongation-competent nuclei (P (0) ≠ 0), their kinetics diverge over time as monomer depletion and *de novo* P formation proceed differently (compare Figures 2H,M). A combined scenario in which the inhibitor binds both m and P is also considered and described in Supplementary Figure S2.

It is important to highlight that in both kinetic models presented in Figures 1E, 2I, which involve formation of mI state, we have excluded further dimerization (mI)_2_, tetramerization (mI)_4_ or heterotypic associations (e.g., m_2_I, mI_2_), possibilities previously described for htt^ex1^Q_7_ in the presence of SH_3_ protein inhibitors (Ceccon et al., 2021). While a fully generalized model is theoretically possible, it would require many speculative parameters that are not currently constrained by experimental data.

Here, we treat mI as an off-pathway species that sequesters aggregation-competent monomers and suppresses downstream oligomer formation. This approach contrasts with a recent study by Knowles and Vendruscolo (Habchi et al., 2017) which investigated conditions where the m ⇌ mI equilibrium is strongly shifted toward mI. Under such regime, aggregation kinetics are reinterpreted based on the behavior of the mI species itself, effectively treating it as an alternative aggregation-competent species with its own nucleation and elongation characteristics.

In our system, however, full saturation of the m ⇌ mI equilibrium is generally unachievable due to two main limitations: (a) the limited aqueous solubility of polyphenolic inhibitors (in the low mM range) and (b) the micromolar-range dissociation constant (*K*_d_ ∼ μM). As a result, monomer and mI species coexist, leading to a complex inhibition landscape in which multiple pathways overlap and contribute to the observed behavior.

## 3 Conclusions and perspective

Advanced solution-state NMR, especially relaxation-based techniques, provides a unique window into the fleeting early stages of amyloid aggregation—critical for effective therapeutic intervention. Building on established NMR methods and kinetic modeling of htt^ex1^ aggregation, we demonstrate that NMR combined with kinetic analysis can distinguish oligomerization pathways, define inhibitor binding modes, and classify inhibition mechanisms. This mechanistic insight goes beyond traditional endpoint assays, enabling accurate, pathway-specific screening of natural compounds. We advocate a paradigm shift toward NMR-based monitoring of aggregation inhibition, a strategy that accelerates the discovery of disease-modifying agents with well-defined molecular modes of action and supports the integration of natural compounds into therapeutic and nutritional frameworks. Polyphenols emerge as versatile and tunable scaffolds, offering significant potential for rational anti-amyloid drug design. Importantly, investigating the effects of polyphenols on amyloid aggregation holds broad implications across nutritional science, biomedicine, and preventive health, given their dietary accessibility and bioactivity.

## Data Availability

The raw data supporting the conclusions of this article will be made available by the authors, without undue reservation.
