# Supplementary material for: Molecular mechanisms of amyloid inhibition: an NMR-driven framework with polyphenols as a case study
Source: Front Mol Biosci. 2025 Sep 12;12:1676927. doi: 10.3389/fmolb.2025.1676927 (PMC12463619; doi:10.3389/fmolb.2025.1676927)
Supplement: Supplementary file 1 [file Supplementaryfile1.docx]

**Supplementary information**

**Molecular mechanisms of amyloid inhibition: an NMR-driven framework with polyphenols as a case study**

Giacomo Zuccon^1,2^, Aakriti Darnal^1^, Edoardo Longo ^2^, Sara D’Aronco ^2^, Emanuele Boselli ^2^, Patrick Orlando^3^, Alberto Ceccon^1,*^

1. Laimburg Research Centre, Laimburg 6 - Pfatten (Vadena), 39040 Auer (Ora), BZ, Italy.
2. Faculty of Agricultural, Environmental and Food Sciences, Free University of Bozen-Bolzano, Piazza Università 5, 39100 Bozen-Bolzano, Italy.
3. Department of Life and Environmental Sciences, Polytechnic University of Marche, Ancona 60131, Italy

**Kinetic Models of Amyloid Aggregation with Inhibition**

Building upon the unified kinetic model of *htt^ex1^* pre-nucleation and fibril formation proposed by the group of G. Marius Clore (Torricella et al., 2024), we introduce an extension that incorporates the effects of small-molecule inhibitors acting at multiple stages of the aggregation process.

In this extended model, a small inhibitor molecule (denoted **I**) is introduced. This molecule can bind reversibly to both the free monomer (**m**) and/or the extendable fibril ends (**P**). The binding of **I** to either **m** or **P** is assumed to occur on a timescale significantly faster than the aggregation process. Therefore, the inhibitor-binding reactions can be treated under a rapid equilibrium approximation (quasi-instantaneous relative to the slower aggregation kinetics).

The time evolution of fibril formation is described by the following system of coupled differential equations:

$\frac{dP}{dt}=k_{c}[T\left( t \right)]+k_{2}[{m^{free}(t)]}^{n2}M(t)$ (S1)

$\frac{dM}{dt}=2k_{+}[m^{free}\left( t \right)]P^{free}(t)$ (S2)

Where $T(t)$ is the time-dependent concentration of tetramers, M(t) is the fibril mass (expressed in monomer units), and the time-dependent concentration of available “free” monomer (not bound to the inhibitor) is given by $m^{free}\left( t \right)$ = m(*t*) – mI(*t*), with m(*t*) = m^tot^ − M(*t*). Here, m^tot^ is the total monomer concentration, and mI(*t*) represents the time-dependent concentration of monomer-inhibitor complex.

Similarly, the time-dependent concentration of “free” (not bound to the inhibitor) extendable fibril ends is obtained as $P^{free}\left( t \right)$= P(*t*) – PI(*t*), where P(*t*) is the total number concentration of fibril ends, and PI(*t*) is the concentration of extendable ends bound to the inhibitor.

Importantly, although both mechanisms initially produce similar inhibition profiles due to pre-existing elongation-competent nuclei (P_0_ ≠ 0), their kinetics diverge over time as monomer depletion and *de novo* P formation proceed differently (compare Fig. 2H and 2M). A combined scenario in which the inhibitor binds both m and P is also considered and described in Fig. S2.

The rate constants $k_{c}$, $k_{2}$, and $k_{+}$ in (S1) and (S2) are tetramer conversion to “active nuclei” (in units of h^-1^), secondary nucleation (in units of M^-n2^ h^-1^, where n2 is the corresponding order of the reaction), and elongation (in units of M^-1^ h^-1^), respectively.

At any given time, the concentration of “free” ligand ($I^{free}$) can be obtained as follows:

${[I]}^{free}={[I]}^{\mathrm{tot}}-\left[ \mathrm{mI} \right]-[PI]$ (S3)

Given the equilibrium between “*free*” monomer and inhibitor: $m+I\leftrightarrow mI$, the equilibrium dissociation constant ($K_{D,mI})$ can be obtained as:

$K_{D,mI}=\frac{{[m]}^{free}\left[ I \right]}{\left[ \mathrm{mI} \right]}=\frac{\left( \left[ m \right]-\left[ \mathrm{mI} \right] \right)\left( {[I]}^{\mathrm{tot}}-\left[ \mathrm{mI} \right]-\left[ \mathrm{PI} \right] \right)}{\left[ \mathrm{mI} \right]}$ (S4)

Note that the superscript “*free*” on [m] is explicitly included in Eq. S4 to avoid confusion with the total monomer concentration available participating in aggregation, m(t) = m^tot^ − M(t).

Since $\left[ \mathrm{PI} \right]<<\left[ \mathrm{mI} \right]$ throughout the aggregation reaction (Fig. S1), we approximate the “*free*” inhibitor concentration as $\left[ I \right]\approx I^{\mathrm{tot}}-\left[ \mathrm{mI} \right]$. This leads to a quadratic equation in [mI], which can be solved independently of [PI], leading to:

$[mI]=\frac{\left( [m]+K_{D,mI}+{[I]}^{\mathrm{tot}} \right)-\sqrt{\left( [m]+K_{D,mI}+{[I]}^{\mathrm{tot}} \right)^{2}-4[m]{[I]}^{\mathrm{tot}}}}{2}$ (S5)

The concentration of “*free*” monomer that enters S1 is $\left[ m \right]^{free}\left( t \right)=m^{\mathrm{tot}} - M\left( t \right)-[mI] (t)$.

Similarly, the equilibrium $P+I\leftrightarrow PI$is described by the following equilibrium dissociation constant ($K_{D,PI})$:

$K_{D,PI}=\frac{{[P]}^{free}\left[ I \right]}{\left[ PI \right]}=\frac{\left( \left[ P \right]-\left[ PI \right] \right)\left( {[I]}^{tot}-\left[ mI \right]-\left[ PI \right] \right)}{\left[ PI \right]}$ (S6)

From which, $[PI]=\frac{\left( \left[ P \right]\left[ I \right]^{\mathrm{tot}}-\left[ P \right]\left[ \mathrm{mI} \right] \right)}{K_{D,PI}+\left[ I \right]^{\mathrm{tot}}-\left[ \mathrm{mI} \right]}$ (S7)

The concentration of free elongation-competent nuclei that enters S2 is thus:

$\left[ P \right]^{free}(t)=P(t)-[\mathrm{mI}] (t)$ (S8)

Assuming a highly skewed equilibrium distribution ($p_{D},p_{T}\ll p_{m}$) , the time-dependent tetramer concentration, $[T(t)]$, can be related to the time-dependent concentration of available monomer as: $\left[ T\left( t \right) \right]={{[m\left( t \right) ]}^{4}}/{(K_{D1}^{2}}K_{D2})$, where $K_{D1},K_{D2}$ are the equilibrium dissociation constants for the step m ⇌ D and D ⇌ T, respectively.

The optimized values of the rate constants as obtained from the global fit of time-dependence intensity decay (in the absence of inhibitor, as reported in our previous publication (Ceccon et al., 2022) as shown in Fig. 2E and listed in Table S1. Note that the value for $k_{c}$ was recast from the following relationship: $k_{c}= k_{n}K_{D1}^{2}K_{D2}$ as described in (Torricella et al., 2024).

**Simulation of NMR intensity decay due to aggregation of htt^ex1^Q_35_ in the presence and absence of inhibitor.**

The NMR signal intensities of htt^ex1^Q_35_ $\left( I_{calc} \right)$ as in Fig. 2E, H, M are calculated from:

$I_{calc}=\left. 1-\alpha M\left( t \right)/m_{tot} \right.$ (S9)

Where α (= 0.896) is a scaling factor accounting for the fact that NMR intensities for the PRD do not decay to zero for fully aggregated samples, M(t) is the (mass) concentration of the fibrils as defined in eq. S2 and m^tot^ (= 0.375 mM) is the total htt^ex1^Q_35_ concentration at t = 0 h.

As noted in the main test, a small amount of residual seeding is inevitably present in the sample, such as P(*0*) ≠ 0. The initial seed will enter eq. S2 in the calculation of P_free_(t). Throughout all simulations, we used P(*0*) = 1.49 × 10^−8^ M, which corresponds to the residual seeding associated with the starting monomer concentration (m^tot^ = 0.375 mM) as indicated in (Ceccon et al., 2022)

**Simulation of ^15^N/^13^Cα-exchange-induced chemical shifts (δ_ex_) and ^15^N/^13^Cα-CPMG relaxation dispersion data for htt^ex1^Q_7_ in the presence of an inhibitor.**

To investigate the influence of inhibitor binding on early-stage oligomerization events of the huntingtin exon 1 fragment (htt^ex1^Q_7_), we implemented a kinetic and relaxation model based on the reversible transitions between five molecular states. The system comprises the monomeric species (m), an off-pathway dimer (D*), an on-pathway dimer (D), an on-pathway tetramer (T), and the inhibitor-bound monomer complex (mI). The model tracks how these species populate under equilibrium conditions, and how their interconversion dynamics give rise to NMR observables (δ_ex_, R_2,eff_).

The simulation is based on a solution of a five-state kinetic scheme where the species interconvert via the reversible processes illustrated in Fig. 1C. All parameters of exchange {$k_{1};k_{-1};k_{2};k_{-2};k_{3};k_{-3}\}$, transverse and longitudinal relaxation rates {$R_{2,m}$= 6.4 s^-1^; with $R_{2,T}={2*R}_{2,D*}$=${2*R}_{2,D}$= ${4*R}_{2,m}$ and $R_{1,m}$ = 1.5 s^-1^ with $R_{1,T}=R_{1,D*}$=$R_{1,D}$= $R_{1,m}$} and chemical shift values for Lys8 {$\Delta_{\omega,D*}$= -1.15 ppm, $\Delta_{\omega,D}$= $\Delta_{\omega,T}$= -3.33 ppm} were obtained from previous NMR studies on htt^ex1^Q_7_ by Clore and coworkers (Ceccon et al., 2020; Clore, 2022).

We assumed a dissociation constant *K*_D,mI_ = 50 μM for the equilibrium, $m+I\leftrightarrow mI,$ consistent with previously reported literature on the binding of polyphenols to monomeric amyloidogenic proteins which suggests low micromolar affinities (Ahmed et al., 2017; Marcinko et al., 2020). The assumed off-rate k_-4_ and chemical shift difference $\Delta_{\omega,mI}$ are 2000 s^-1^ and - 0.1 ppm, respectively.

The simulation proceeds by evaluating a range of total monomer htt^ex1^Q_7_ concentrations (m^tot^ spanning 1 μM to 1.2 mM). For each concentration, the code solves for the “free” inhibitor concentration *I* using the quadratic binding equation for a simple 1:1 complex, and from this computes the fractional populations of all five states under equilibrium conditions. These populations (p_m_, p_D*_, p_D_, p_T_, p_mI_) are calculated analytically. The population of the main (observable) state *m* at each concentration was calculated from the rate constants using the relationship,

$p_{m}=\frac{1}{\left[ 1+\frac{k_{4}^{app}}{k_{-4}}+\frac{k_{3}^{app}}{k_{-3}}+\frac{k_{1}^{app}}{k_{-1}}\left( 1+\frac{k_{2}^{app}}{k_{-2}} \right) \right]}$ (S10)

The remaining populations are given by:

$p_{D*}=p_{m}\left( k_{3}^{app}/k_{-3} \right)$ (S11)

$p_{D}=p_{m}\left( k_{1}^{app}/k_{-1} \right)$ (S12)

$p_{T}=p_{D}\left( k_{2}^{app}/k_{-2} \right)$ (S13)

$p_{mI}=p_{m}\left( k_{4}^{app}/k_{-4} \right)$ (S14)

Where the phenomenological (“apparent”), pseudo-first-order forward rate constants $k_{i}^{app}\left( i\in\left\{ 1,2,3,4 \right\} \right)$are related to the corresponding ‘true’ kinetic rate $k_{i}$ constants through,

$k_{1}^{app}=2k_{1}\left[ m \right]^{free}$ (S15)

$k_{3}^{app}=2k_{3}\left[ m \right]^{free}$ (S16)

for dimerization,

$k_{2}^{app}=2k_{2}\left( \frac{k_{1}}{k_{-1}} \right)\left( \left[ m \right]^{free} \right)^{2}$ (S17)

for tetramerization, and

$k_{4}^{app}=k_{4}\left[ I \right]^{free}$ (S18)

for monomer-inhibitor binding, where $\left[ m \right]^{free}$ obtained from S5. Note that since the population of the oligomeric states (D and T) is small (< 5 %) under the experimental conditions employed, in the calculation *of* $\left[ m \right]^{free}$, [D], [D*], and [T] can be neglected.

The Liouvillian that enters the sets of Bloch-McConnell equations analyzed can be represented by the matrix $\tilde{R}$ = $\tilde{R}^{CS}$+ $\tilde{R}^{rel}$ + $\tilde{R}^{ex}$ where $\tilde{R}^{CS}$ is a diagonal matrix of the differences in chemical shifts (Δω) of each state with respect to the main, observable state (m), $\tilde{R}^{rel}$ is a diagonal matrix of transverse spin relaxation rates (R_2_) of each state, and $\tilde{R}^{ex}$ is the matrix describing the kinetics of chemical exchange between different states. When the inhibitor is added to labeled htt^ex1^Q7,

$\tilde{R}^{CS}=-i\left[ \begin{matrix} 0 & 0 & 0 & 0 & 0 \\ 0 & {\Delta\omega}_{D^{*}} & 0 & 0 & 0 \\ 0 & 0 & {\Delta\omega}_{D} & 0 & 0 \\ 0 & 0 & 0 & {\Delta\omega}_{T} & 0 \\ 0 & 0 & 0 & 0 & {\Delta\omega}_{mI} \end{matrix} \right]$ (S19)

$\tilde{R}^{rel}=-\left[ \begin{matrix} R_{2,m} & 0 & 0 & 0 & 0 \\ 0 & R_{2,D*} & 0 & 0 & 0 \\ 0 & 0 & R_{2,D} & 0 & 0 \\ 0 & 0 & 0 & R_{2,T} & 0 \\ 0 & 0 & 0 & 0 & R_{2,mI} \end{matrix} \right]$ (S20)

$\tilde{R}^{ex}=-\left[ \begin{matrix} k_{3}^{app}+k_{1}^{app}+k_{4}^{app} & -k_{-3} & -k_{-1} & 0 & -k_{-4} \\ -k_{3}^{app} & k_{-3} & 0 & 0 & 0 \\ -k_{1}^{app} & 0 & k_{-1}+k_{2}^{app} & -k_{-2} & 0 \\ 0 & 0 & -k_{2}^{app} & k_{-2} & 0 \\ {-k}_{4}^{app} & 0 & 0 & 0 & k_{-4} \end{matrix} \right]$ (S21)

where ${\Delta\omega}_{k}$ $\left( k\in\left\{ m,D^{*},D,T,mI \right\} \right)$is the difference between chemical shifts of states *k* and the state *m* in rad/s (with $\omega_{m}$= 0 ppm); R_2,k_  $\left( k\in\left\{ m,D^{*},D,T,mI \right\} \right)$are the transverse relaxation rate of states *k* in the absence of exchange. In the exchange matrix, $k_{i}^{app}$ are pseudo first-order apparent association constants calculated as previously described.

Exchange-induced chemical shifts, δ_ex_ (in Hz), were calculated from the imaginary part of the smallest (by absolute magnitude) eigenvalue of the matrix R, δ_ex_ = *Im*(min[eig{$\tilde{R}$}])/2π, where eig{$\tilde{R}$} is a vector of complex eigenvalues of $\tilde{R}$. In Fig. 1D (left panel) δ_ex_ data were simulated as a function of the concentration of htt^ex1^Q_7_ and in the presence of different amounts of inhibitor (0.0 mM < [I] < 0.8 mM).

CPMG relaxation dispersion profiles obtained for isotopically labeled htt^ex1^Q_7_ in the presence of the inhibitor, monitor the changes in effective transverse relaxation rates, R_2,eff_, as a function of CPMG frequency, υ_CPMG_. The evolution of magnetization during the constant-time CPMG relaxation period is given by, $\vec{M}(t)= {(AA^{*}A^{*}A)}^{n}M(0)$where the column vector $\vec{M}$ is [M_m_ M_D*_ M_D_ M_T_ M_mI_]^T^; A = exp(-$\tilde{R}$τ_cp_/2), A* is complex conjugate of A, τ_cp_ is the distance between successive 180° pulses, and *n* the number of CPMG cycles. All the states were assumed to be present at the start of the CPMG train, M(0) = (p_m_, p_D*_, p_D_, p_T_, p_mI_). The rates R_2,eff_ are calculated from the decay of the magnetization of the main, observable species for each υ_CPMG_ (number of CPMG cycles *n*). In Fig. 1D (left and right panel) ^15^N - R_2,eff_ were simulated for [htt^ex1^Q_7_] = 0.8mM in the presence of different amounts of inhibitor (0.0 mM < [I] < 0.8 mM).

**Simulation of concentration-dependent ^15^N-R_1ρ_ measurements in the presence of the inhibitor**

In-phase ^15^N-R_1ρ_ were simulated by solving the set of homogeneous Bloch-McConnell equations (McConnell, 1958) for the exchanging system shown in Fig. 1E via the propagation of a 15x15 dimensional Liouvillian $\tilde{L}$ given by,

$\tilde{L}_{12}=\left[ \begin{matrix} \tilde{R}_{m} & 0 & 0 & 0 & 0 \\ 0 & \tilde{R}_{D*} & 0 & 0 & 0 \\ 0 & 0 & \tilde{R}_{D} & 0 & 0 \\ 0 & 0 & 0 & \tilde{R}_{T} & 0 \\ 0 & 0 & 0 & 0 & \tilde{R}_{mI} \end{matrix} \right]+\tilde{R}^{ex}\otimes\tilde{I}_{3}$ (S22)

where $\tilde{R}^{ex}$is the exchange matrix (Eq. S21), $\tilde{I}_{3}$is the 3-dimensional identity matrix, ‘⊗ ’ denotes the Kronecker product, and each of the 3x3 matrix blocks $\tilde{R}_{k}$ of the block-diagonal matrix on the right-hand side of Eq. S22 (where k is a particular state, $k\in\left\{ m,D^{*},D,T,mI \right\}$), has the form,

$\tilde{R}_{k}=\left[ \begin{matrix} R_{2,k} & \Omega^{k} & 0 \\ -\Omega^{k} & R_{2,k} & \omega_{1} \\ 0 & -\omega_{1} & R_{1,k} \end{matrix} \right]$ (S23)

where $R_{1,k}$ and $R_{2,k}$are intrinsic longitudinal and transverse spin relaxation rates, respectively, of state *k* in the absence of exchange; $\Omega^{k}$ are the resonance frequency of state *k* (in rad/sec), and $\omega_{1}$ is the strength of the continuous-wave irradiation (‘spinlock’) RF field (= 1500 Hz) applied along the x-axis. Note that in Fig. 1D, in-phase ^15^NR_1ρ_ were simulated as a function of concentration of htt^ex1^Q_7_ and in the presence of different amounts of inhibitor (0.0 mM < [I] < 0.8 mM).

**Simulation of the concentration-dependent exchange-induced chemical shifts (δ_ex_) and V/I ratios together with the aggregation profiles of htt^ex1^Q_35_**

Concentration-dependent calculation of ^15^N-**δ**_ex_, cross-peak volume/intensity (V/I) ratios and simulation of time-dependent aggregation profiles of htt^ex1^Q_35_ followed the procedures previously described (Ceccon et al., 2022).

The Liouvillian matrix $\tilde{R}$, describing the evolution of the magnetization for the exchange system mI ↔ m ↔ D ↔ T (notice we are not considering D* in line with the previous study) is given by $\tilde{R}$ = $\tilde{R}^{CS}$+ $\tilde{R}^{rel}$ + $\tilde{R}^{ex}$ where $\tilde{R}^{CS}=\left( 0 0 0 0, 0 {\Delta\omega}_{D} 0 0, 0 0 {\Delta\omega}_{T} 0, 0 0 0 {\Delta\omega}_{\mathrm{mI}} \right),$ $\tilde{R}^{rel} =\left( R_{2,m} 0 0 0, 0 R_{2,D} 0 0, 0 0 R_{2,T} 0, 0 0 0 R_{2,mI} \right)$ and $\tilde{R}^{ex} =-\left( k_{1}^{app}+k_{4}^{app} -k_{-1} 0 -k_{-4} , -k_{1}^{app} k_{-1}+k_{2}^{app} -k_{-2} 0, 0 -k_{2}^{app} k_{-2} 0, {-k}_{4}^{app} 0 0 k_{-4} \right).$ δ_ex_ values in Fig. 2 D,G (right panels) were calculated from the imaginary part of the smallest (by absolute magnitude) eigenvalue of the relaxation matrix $\tilde{R}$: δ_ex_ (Hz) = Im(min[eig{$\tilde{R}$}])/2π, where eig{$\tilde{R}$} is a vector of complex eigenvalues of R .

Calculation of V/I values in Fig. 2D,G (left panels) followed the description in the supplementary information of Ceccon et al., 2022. The intrinsic ^15^N-^1^H double quantum (^15^N-R_2,DQ_) and zero-quantum (^15^N-R_2,ZQ_) transverse relaxation rates were assumed to be equal to the single-quantum ^15^N-R_2_ values measured experimentally with the “effective” relaxation rates, R_2,eff_ = R_2_ + R_ex_, estimated from the real part of the smallest (by absolute magnitude) eigenvalue of the matrix $\tilde{R}$: R_2,eff_ = -Re(min[eig{$\tilde{R}$}).

The parameters of exchange {$k_{1};k_{-1};k_{2};k_{-2}\},$longitudinal ^15^N- and ^1^H- relaxation rates {^15^N-$R_{2,m}$= 7.5 s^-1^ and ^1^H-$R_{2,m}$= 21.0 s^-1^ with $R_{2,T}={2*R}_{2,D*}$=${2*R}_{2,D}$= ${4*R}_{2,m}$}, ^15^N- and ^1^H- chemical shift values for Lys8 {^15^N- ${\Delta\omega}_{D}$= ^15^N- ${\Delta\omega}_{T}$= -3.33 ppm, ^1^H- ${\Delta\omega}_{D}$= ^1^H- ${\Delta\omega}_{T}$= -0.6 ppm} were taken from previous NMR studies on htt^ex1^Q_35_ by Ceccon et al., 2022.

As in the htt^ex1^Q_7_ case, we assumed *K*_D,mI_ = 50 μM for the equilibrium, $m+I\leftrightarrow mI,$ k_-4_ = 2000 s^-1^, ^15^N- and ^1^H- ${\Delta\omega}_{mI}$ = - 0.1 ppm, respectively.

**Consideration on the behavior of concentration-dependent δ_ex_ measured on htt^ex1^Q_7_ and htt^ex1^Q_35_ in the presence of the inhibitor as shown in Fig. 1B and Fig. 2G**

The assumed parameters for the exchange equilibrium, m + I ⇌ mI are *K*_D,mI_ = 50 μM, k_-4_ = 2000 s-1) and an assumed chemical shift change, ^15^N - $\left| \Delta_{\omega,mI} \right|$ = 0.1 ppm. These parameters place the system in the fast exchange regime on the chemical shift time scale, as $\frac{k_{ex}}{\Delta_{\omega,mI}}\sim\frac{k_{-4}}{\Delta_{\omega,mI}}$ ~ 40.

Note that the chosen value for $\Delta_{\omega,mI}$ is consistent with previous reports on flavonoid–protein interactions, particularly involving disordered domains such as those in htt^ex1^, where backbone amide nitrogen chemical shift changes are typically detectable but relatively small (Ahmed et al., 2017; Huang et al., 2012).

In this fast exchange regime, the observed chemical shift $\delta_{obs}$​ is a population-weighted average of the free (m) and bound (mI) states. At the low htt^ex1^Q_7_ (and htt^ex1^Q_35_) concentrations used in the simulation ([htt^ex1^] ~ < 200 μM)_,_ where the populations of oligomeric species (D*, D, T) can be assumed to be negligible (∼0) the observed chemical shift can be expressed as: $\delta_{obs}=p_{mI}{\Delta\omega}_{mI}$ (note that throughout all simulations $\omega_{m}$ is set at 0 ppm). It follows directly that in the presence of the inhibitor, the value of the y-intercept is ≠ 0 in both Fig. 1B (left panel) and Fig. 2G (left panel). For example, in Fig. 1B (left panel) at [htt^ex1^] = 1 μM, the ^15^N-δ_ex_ values are -6.4 Hz ($p_{mI}$ ~ 79 %), -7.0 Hz ($p_{mI}$ ~ 88 %), -7.5 Hz ($p_{mI}$ ~ 94 %) for inhibitor concentration of [I] = 0.2, 0.4, 0.8 mM, respectively.

Moreover, at a fixed inhibitor concentration, the simulated curves of ^15^N-δ_ex_ as a function of increasing htt^ex1^ concentration show a markedly different behavior compared to the case of [I]=0 mM. Specifically, in Fig. 1B (left panel), ^15^N-δ_ex_ data initially becomes more positive in the range 1 μM <[htt^ex1^Q_7_] < 0.6 mM, before decreasing again for [htt^ex1^Q_7_] > 0.6 mM, and reaching approximately −20 Hz at 1.2 mM. This behavior is directly related to the shifting partitioning among the species m, mI, D*, D, and T. As [htt^ex1^Q_7_] increases, the binding equilibrium between m and mI is progressively outcompeted by the self-association pathway. This leads to re-entry into exchange involving the D and T oligomeric species, which are characterized by a ¹⁵N chemical shift difference of ^15^N- ${\Delta\omega}_{D}$= ^15^N- ${\Delta\omega}_{T}$= -3.33 ppm.

**Table S1.** List of values for the rate constants used in the simulations describing prenucleation tetramerization and primary nucleation/fibrillation of httᵉˣ^1^Q₃₅ as obtained in Ceccon et al., 2022.

| Rate constant | | Values |
| --- | --- | --- |
| *Pre-nucleation* | | |
| k_1_ (M^-1^ s^-1^) | | 6.2 (± 1.8) × 10^5^ |
| k_-1_ (s^-1^) | | 3.8 (± 1.0) × 10^4^ |
| k_2_ (M^-1^ s^-1^) ^a^ | | 1.8 × 10^9^ |
| k_-2_ (s^-1^) ^b^ | | 2 × 10^4^ |
| *Primary nucleation/Fibrillation* | | |
| k_c_ (h⁻¹) ^c^ | 0.014 | |
| k⁺ (M⁻¹ h⁻¹) | 7.8 (±0.3) × 10⁵ | |
| k_S_ (M⁻¹ h⁻¹) | 0.50 ± 0.02 | |
|  |  | |

^a,b^ Note that only lower bounce values for these two rate constants could be established given the impossibility to perform time-extensive ^15^N-R1rho measurements. ^c^As described previously, the value for $k_{c}$ was recast from the following relationship: $k_{c}= k_{n}K_{D1}^{2}K_{D2}$ as described in Torricella et al., 2024 using previously determined k_n_ (M^-3^ h⁻¹) = 3.4 (± 0.4) × 10^5^ in Ceccon et al., 2022.


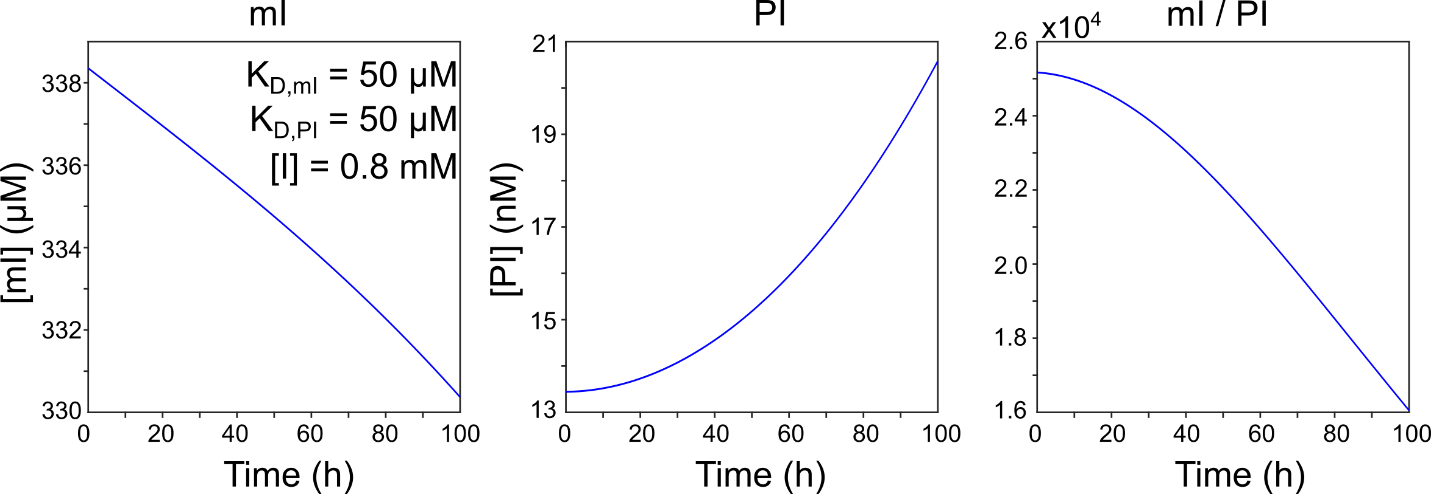


**Figure S1.** Simulated time-dependent concentrations of: (left) the monomeric htt^ex1^Q_35_–inhibitor complex (mI), (middle) extendable fibril ends bound to the inhibitor (PI), and (right) the concentration ratio mI/PI. Simulations were performed for [m] = 0.38 mM, [I] = 0.8 mM and $K_{D,mI}$ = $K_{D,PI}$ = 50 μM.


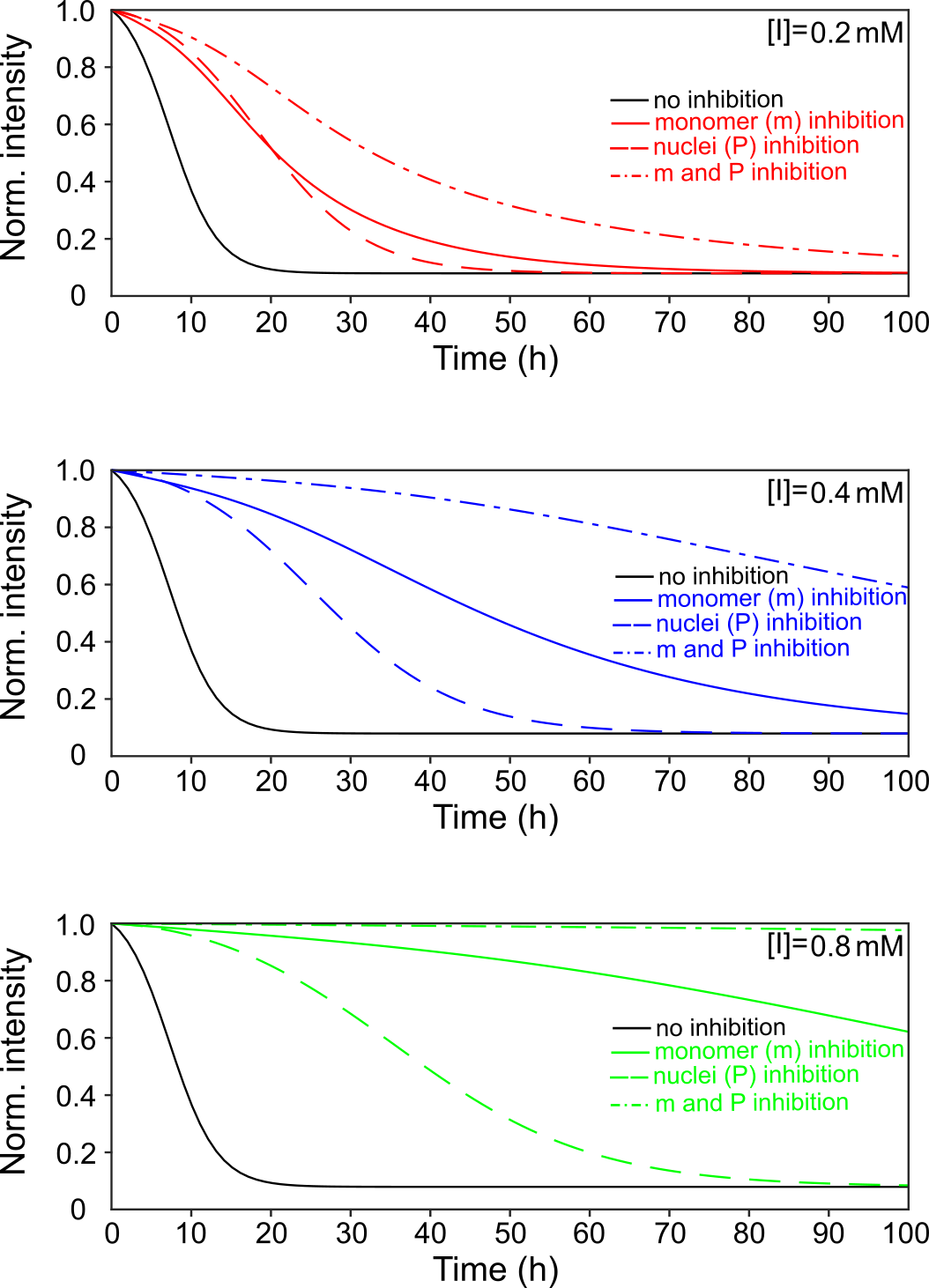


**Figure S2.** Simulated decay of the PRD signal intensity under different inhibition mechanisms. All simulations were performed with [htt^ex1^] = 0.38 mM, $K_{D,mI}$ = $K_{D,PI}$= 50 μM . Each panel corresponds to a different inhibitor concentration: 0.2 mM (top, red curves), 0.4 mM (middle, blue curves), and 0.8 mM (bottom, green curves). Within each panel, the black curve represents signal decay in the absence of inhibition. The three inhibition scenarios simulated are: inhibition of monomeric species m (solid lines), inhibition of elongation-competent nuclei P (dashed lines), and simultaneous inhibition of both m and P (dash-dotted lines).

**References:**

1. Ahmed, R., VanSchouwen, B., Jafari, N., Ni, X., Ortega, J., Melacini, G., 2017. Molecular Mechanism for the (−)-Epigallocatechin Gallate-Induced Toxic to Nontoxic Remodeling of Aβ Oligomers. J. Am. Chem. Soc. 139, 13720–13734. https://doi.org/10.1021/jacs.7b05012
2. Ceccon, A., Tugarinov, V., Clore, G.M., 2020. Kinetics of Fast Tetramerization of the Huntingtin Exon 1 Protein Probed by Concentration-Dependent On-Resonance *R*_1ρ_ Measurements. J. Phys. Chem. Lett. 11, 5643–5648. https://doi.org/10.1021/acs.jpclett.0c01636
3. Ceccon, A., Tugarinov, V., Torricella, F., Clore, G.M., 2022. Quantitative NMR analysis of the kinetics of prenucleation oligomerization and aggregation of pathogenic huntingtin exon-1 protein. Proc. Natl. Acad. Sci. U.S.A. 119, e2207690119. https://doi.org/10.1073/pnas.2207690119
4. Clore, G.M., 2022. NMR spectroscopy, excited states and relevance to problems in cell biology – transient pre-nucleation tetramerization of huntingtin and insights into Huntington’s disease. Journal of Cell Science 135. https://doi.org/10.1242/jcs.258695
5. Huang, R., Vivekanandan, S., Brender, J.R., Abe, Y., Naito, A., Ramamoorthy, A., 2012. NMR Characterization of Monomeric and Oligomeric Conformations of Human Calcitonin and Its Interaction with EGCG. Journal of Molecular Biology 416, 108–120. https://doi.org/10.1016/j.jmb.2011.12.023
6. Marcinko, T.M., Drews, T., Liu, T., Vachet, R.W., 2020. Epigallocatechin-3-gallate Inhibits Cu(II)-Induced β-2-Microglobulin Amyloid Formation by Binding to the Edge of Its β-Sheets. Biochemistry 59, 1093–1103. https://doi.org/10.1021/acs.biochem.0c00043
7. McConnell, H.M., 1958. Reaction Rates by Nuclear Magnetic Resonance. The Journal of Chemical Physics 28, 430–431. https://doi.org/10.1063/1.1744152
8. Torricella, F., Tugarinov, V., Clore, G.M., 2024. Nucleation of Huntingtin Aggregation Proceeds via Conformational Conversion of Pre‐Formed, Sparsely‐Populated Tetramers. Advanced Science 11, 2309217. https://doi.org/10.1002/advs.202309217
